# Obstructive Giant Inflammatory Polyposis: Is It Related to the Severity or the Duration of the Inflammatory Bowel Disease? Two Case Reports

**DOI:** 10.1155/2018/3251549

**Published:** 2018-06-10

**Authors:** Antoine Abou Rached, Jowana Saba, Leila El Masri, Mary Nakhoul, Carla Razzouk

**Affiliations:** ^1^Lebanese University, School of Medicine, Lebanon; ^2^Saint-Joseph University, School of Medicine, Lebanon

## Abstract

We report two cases of giant inflammatory polyposis (GIP) with totally different presentation and evolution. The first patient had two giant pseudopolyps after one year of the diagnosis of UC. The second patient had one obstructive giant pseudopolyp secondary to CD at the level of the transverse colon, being totally asymptomatic years before the presentation. GIP is a rare complication of inflammatory bowel disease (IBD). It consists of numerous filiform polyps that look like a “mass of worms” or a “fungating” mass. Surgical resection is inevitable when GIP presents with obstructive symptoms.

## 1. Introduction

Giant inflammatory polyposis (GIP) is a rare complication of inflammatory bowel disease (IBD) [1]. It occurs more frequently in ulcerative colitis (UC) than in Crohn's disease (CD) [2–4]. We report here two cases of obstructive GIP with totally different presentation and evolution. The first patient had two giant pseudopolyps after one year of the diagnosis of UC. The second patient had one obstructive giant pseudopolyp secondary to CD at the level of the transverse colon, being totally asymptomatic years before the presentation.

## 2. Case Report

### 2.1. Case 1

**Figure 1:**
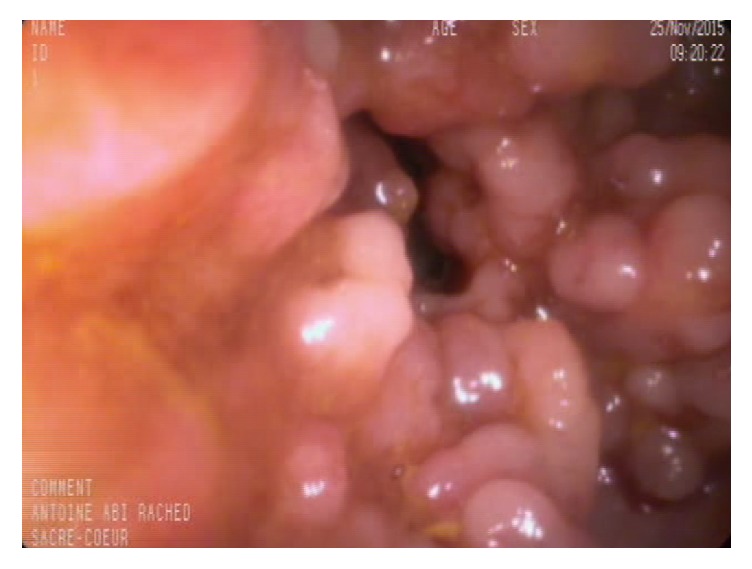
GIP of left colon.

**Figure 2:**
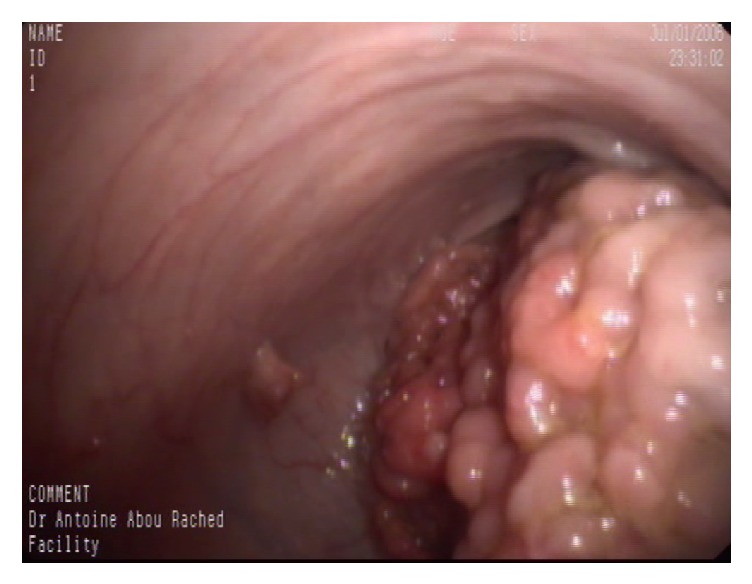
Obstructive GIP of the splenic flexure.

**Figure 3:**
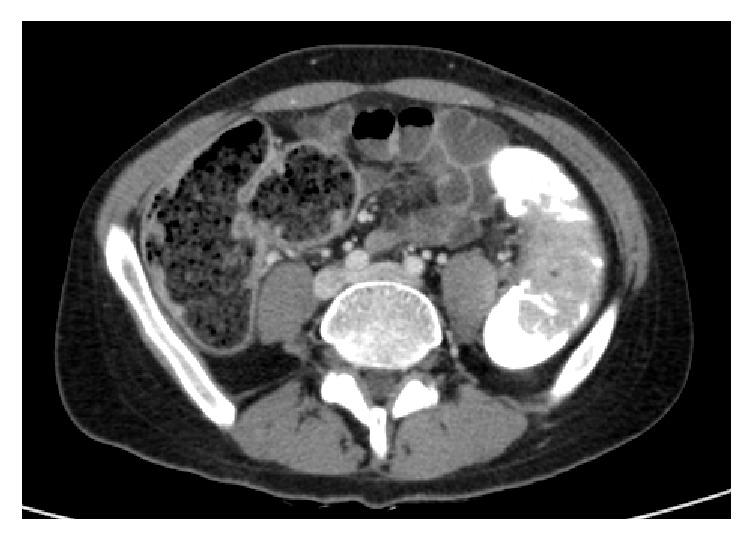
Abdominal CT scan showing GIP the left colon.

**Figure 4:**
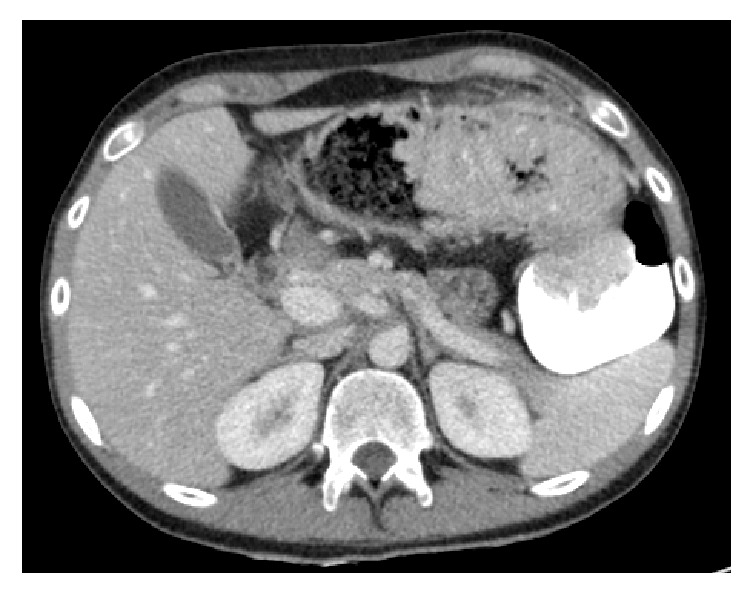
Abdominal CT scan showing obstructive GIP of the splenic flexure.

**Figure 5:**
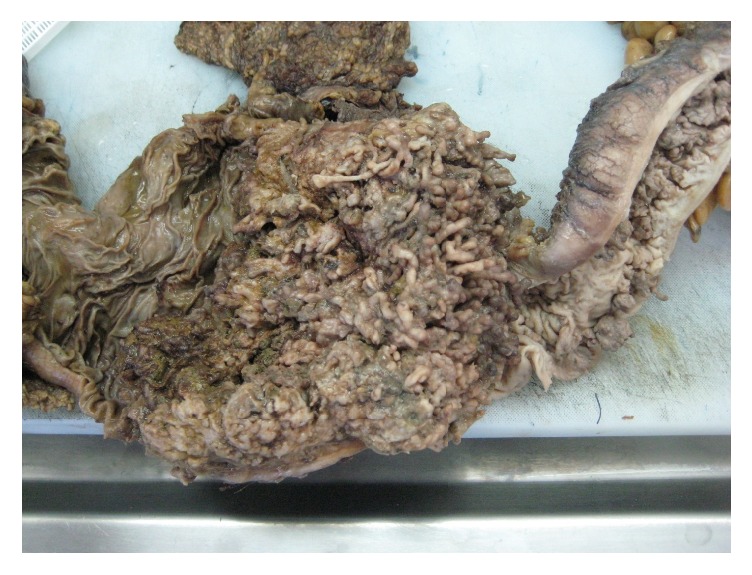
Surgical resection of GIP.

A 20-year-old male presented with worsening bloody diarrhea of 4 months' duration associated with cramping abdominal pain and weight loss of 4 Kg. On admission, he was hemodynamically stable. Physical examination showed mild tenderness to deep palpation in the left lower quadrant. Laboratory tests were consistent with anemia (hemoglobin = 10.5 mg/dl, hematocrit = 33.5%), thrombocytosis (platelets = 568000/mm^3^), low iron level (iron = 25mg/dl), and normal C-reactive protein (CRP). Stool analysis, ova and parasite test,* Clostridium difficile* toxin assay, and stool culture were negative. Colonoscopy revealed left-sided colitis with marked erythema, absent vascular pattern, and friability erosions (Mayo score 2). Biopsies showed chronic active colitis consistent with UC. Based on the clinical presentation and laboratory, endoscopic, and pathologic findings, the patient was diagnosed with moderate left-sided UC and was started on oral and topical 5-aminosalicylic acid (5-ASA) without any response to treatment: bloody diarrhea (more than 5 bowel movement per day), severe abdominal pain, low grade fever, and additional weight loss in addition to severe anemia (hemoglobin = 7.3g/dl) and high CRP with negative stool tests. High dose steroids therapy was started with marked improvement. Steroid tapering caused recurrence of symptoms and anemia at 20mg prednisone per day. Relying on the findings above, the patient had left-sided UC and is steroid-dependent, so Infliximab 5mg/kg was initiated at 0, 2, and 6 weeks, then every 8 weeks without any improvement after 4 months of treatment with persistent bloody diarrhea and severe iron deficiency anemia. Repeated colonoscopy showed severe inflammatory mucosa with deep ulcerations and pseudopolyps formation at the splenic flexure and the distal part of the left colon, separated by healed mucosa. Biopsies from the pathologic area revealed severe chronic active colitis consistent with inflammatory bowel disease/UC without any evidence of cytomegalovirus inclusions. Given the lack of response to Infliximab and the worsening endoscopic findings, resistance to therapy was suspected. Additional laboratory tests showed low Infliximab through level with high antibodies level to Infliximab. The patient was switched to combination therapy with Adalimumab 160mg at week 0, 80mg at week 2, and 40mg every other week plus Azathioprine 2.5mg/Kg with only partial response: improvement of diarrhea but persistence of severe nocturnal colicky abdominal pain. Adalimumab trough level was very low with the absence of anti-drug antibodies. So, Adalimumab was increased to 40mg per week. Two months later, he continued to have severe colicky abdominal pain and distension with weight loss. New colonoscopy revealed complete mucosal healing and a giant pseudopolyp in the left colon and another obstructive one at the level of the splenic flexure (Figures 1 and 2). Biopsies were consistent with chronic inflammation with architectural distortion and cryptic abscess. Abdominal CT scan confirmed the presence of two giant pseudopolyps with evidence of obstruction at the splenic flexure (Figures 3 and 4). The patient had total colectomy (Figure 5) with ileoanal anastomosis and J pouch.

### 2.2. Case 2

**Figure 6:**
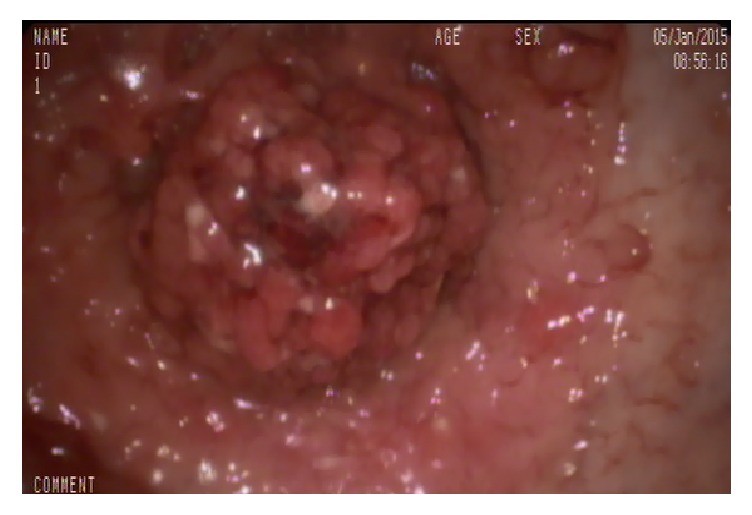
GIP of the transverse colon.

**Figure 7:**
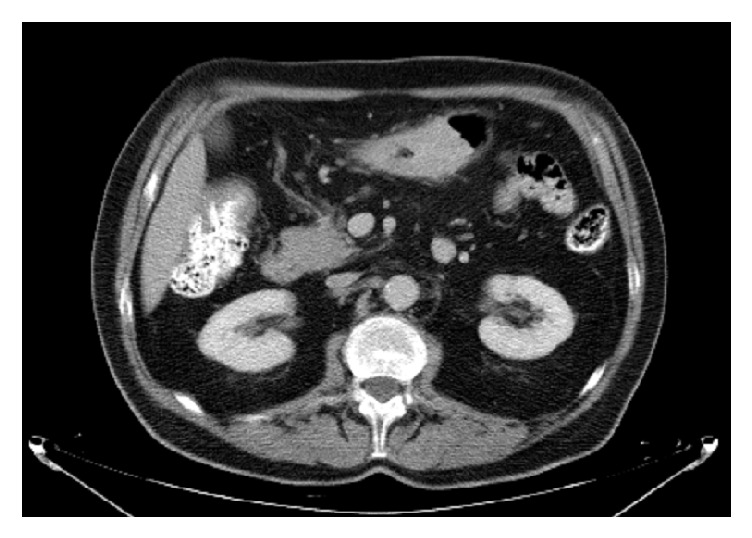
Abdominal CT scan showing stenotic GIP.

A 71-year-old male, previously healthy, was seen for the first time in May 2011 for diarrhea and rectal bleed. His physical examination was unremarkable. Laboratory tests were within normal range. Ileocolonoscopy showed mucosal inflammation and ulcerations over a segment of 7cm at the level of transverse colon. Biopsies were in favor of chronic active colitis. The patient was treated as colonic IBD and was started on Mesalamine 4g per day but he was lost to follow-up. Four years later, he was seen again in January 2015 for the same previously described symptoms. He stated that he took Mesalamine for 6 months and stopped by his own after marked improvement and he was asymptomatic since then until the reappearance of symptoms associated with abdominal pain few days prior to the presentation. Physical examination and lab tests were normal. Colonoscopy revealed an obstructive giant pseudopolyp (Figure 6) at the level of the transverse colon; biopsies showed chronic inflammation with architectural distortion and granulation tissue formation. Abdominal CT scan confirmed the presence of giant pseudopolyp (Figure 7). The patient was treated with segmental colonic resection and the surgical pathologic report was CD. The final diagnosis was colonic CD complicated by an obstructive giant pseudopolyp.

## 3. Discussion

We present two cases of obstructive GIP that developed in two different patients: one with UC and the other with CD. The development of GIP may be related to the severity of the disease in the patient with UC and to the duration of the disease in the patient with CD. In the first case, the patient had UC with severe inflammatory disease leading to the development of two GIP formations, one of them being totally obstructive. In the second, the patient had a quiescent Crohn's colitis disease causing obstructive GIP 5 years after the diagnosis.

GIP is defined by the presence of large polyps that are more than 1.5cm [1, 2]. It consists of numerous filiform polyps that look like a “mass of worms” or a “fungating” mass. It is an uncommon manifestation of inflammatory bowel disease [1], and its presence might be correlated with the severity and the duration of the disease [3]. It occurs most commonly in the transverse colon, followed by the sigmoid and descending colon, the cecum, and the splenic and hepatic flexure [5]. It is associated twice as often in UC compared to CD [2–4]. Yada S et al. reported that the time from the diagnosis of UC to GIP formation ranges from 3 to 276 months [6].

GIP pathogenesis results from repeated peristalsis and fecal stream causing enlarged mucosal tags [5]. Others propose that GIP is formed when inflamed colonic mucosa heals in a polypoid configuration during the regenerative phase, and this happens when ulcerated mucosa becomes surrounded by granulation tissue [3, 4].

The histopathological features include inflammatory infiltrates overlying the muscularis mucosae, deep fissure-like ulcers, chronic mucosal inflammation with lymphoid hyperplasia, and nerve hyperplasia in the surrounding mucosa [6–11].

In a systematic review of literature, Maggs et al. identified 81 GIP formations in 78 patients with IBD. In those with ulcerative colitis, the majority (70%) had extensive colitis and none had proctitis. GIP formations were located throughout the colon although the majority was in the transverse and descending colon [3].

GIP can produce symptoms similar to IBD, such as bloating, diarrhea, discomfort, rectal bleed, abdominal pain, and palpable abdominal mass [3, 12]. 15% were complicated with obstruction and subobstruction and 3% with intussusception of mechanical etiology due to the large size [3]. Others rare complications were noted like protein-losing enteropathy, bleeding, and iron deficiency anemia [13].

GIP is clinically, endoscopically, and radiologically similar to neoplastic lesions and is frequently mistaken for one another [14–19]. The differential diagnosis of GIP includes villous adenoma, dysplasia associated lesion or mass, polypoid carcinoma, lymphoma, and colitis cystica profunda [14–16]. There has only been one reported case of an occult carcinoma arising in a patient who was diagnosed with localized GIP [9]. The incidence of malignancy in a patient with UC is 3–5%, indicating that the risk of malignancy is equal in a patient having UC with or without GIP [9].

Surgical resection is inevitable when GIP presents with obstructive symptoms such as luminal obliterations and/or intussusceptions or they cannot be removed by polypectomy. Extension, chronicity, and acute complications make total coloproctectomy with ileoanal anastomosis the most reasonable therapeutic choice. Some have performed local excision of the pseudopolyp in a bowel-sparing method [20].

In conclusion, we report here two rare cases of GIP associated with UC and CD. The first patient had a dramatic evolution of his UC with the development of two GIP formations within one year of the diagnosis of IBD, one of them being totally obstructive at the splenic flexure, the least common site of GIP. The second patient had CD was totally asymptomatic prior to the development of obstructive GIP.
